# The Role of Repeated Exposure to Multimodal Input in Incidental Acquisition of Foreign Language Vocabulary

**DOI:** 10.1111/lang.12085

**Published:** 2014-10-15

**Authors:** Marie-Josée Bisson, Walter J B van Heuven, Kathy Conklin, Richard J Tunney

**Affiliations:** a University of Nottingham; b Loughborough University

**Keywords:** repeated exposures, frequency effects, incidental learning, multimodality, foreign language vocabulary acquisition

## Abstract

Prior research has reported incidental vocabulary acquisition with complete beginners in a foreign language (FL), within 8 exposures to auditory and written FL word forms presented with a picture depicting their meaning. However, important questions remain about whether acquisition occurs with fewer exposures to FL words in a multimodal situation and whether there is a repeated exposure effect. Here we report a study where the number of exposures to FL words in an incidental learning phase varied between 2, 4, 6, and 8 exposures. Following the incidental learning phase, participants completed an explicit learning task where they learned to recognize written translation equivalents of auditory FL word forms, half of which had occurred in the incidental learning phase. The results showed that participants performed better on the words they had previously been exposed to, and that this incidental learning effect occurred from as little as 2 exposures to the multimodal stimuli. In addition, repeated exposure to the stimuli was found to have a larger impact on learning during the first few exposures and decrease thereafter, suggesting that the effects of repeated exposure on vocabulary acquisition are not necessarily constant.

## Introduction

Foreign language (FL) vocabulary acquisition is a challenging task for FL learners because of the large number of words necessary to achieve fluency in a language. In fact, it is estimated that, in order to have enough knowledge to understand authentic written texts, learners need to know approximately 8,000 word families, representing approximately 35,000 words (Nation, 2001; Schmitt, 2008, 2010). While this number is estimated to be lower for the understanding of spoken discourse, it is still more words than can be realistically taught explicitly in an instructional setting (de Groot & van Hell, 2005; Horst, 2005), hence the importance of incidental vocabulary learning.

Researchers have been using the term incidental learning to describe situations where learning was not required, that is, participants were not explicitly asked to learn (e.g., Pellicer-Sánchez & Schmitt, 2010; Williams, 2010). Rather, learning occurred through mere exposure to FL material during a variety of tasks, following which a surprise word recognition or recall test was completed (Horst, 2005; Hulstijn, 2001; Laufer, 2001). For example, in the case of incidental learning of vocabulary from reading, the aim is often to understand the story or to read for pleasure, and a few new words can be acquired along the way. In contrast, in explicit learning paradigms, learners willingly try to commit some information to memory. For example, in explicit language learning instruction, learners are often provided with a FL word along with its native language (NL) translation equivalent and they have to learn to associate them (de Groot & van Hell, 2005).

Another important distinction must be made between incidental and implicit learning. Implicit learning involves learning without intention and without a clear awareness of what has been acquired (Perruchet & Pacton, 2006; Reber, 1989; Rebuschat & Williams, 2011; Williams, 2005, 2009). Although incidental learning has sometimes been described as learning without intention (e.g., Schmitt, 2010; Williams, 2009), researchers have often used this type of paradigm in language classroom settings where presumably the overarching goal is to learn the language and therefore, in those cases, it is unclear whether the learning was intentional or not (see Gass, 1999, for a discussion). Importantly, however, the focus of the learners was often elsewhere, for example, on comprehension, and therefore learning occurred as a byproduct of another task (Hulstijn, 2001; Schmitt, 2010). Furthermore, in contrast to research on implicit learning, researchers using incidental learning paradigms have been more interested in the amount of learning that occurs, rather than the level of awareness of the participants.

There is a lot of variability in the use of the term incidental learning in the literature. However, the current study and the studies reviewed below use incidental learning to describe learning that was not explicitly required and that happened through mere exposure to the language. In addition, unless otherwise specified, participants in the reviewed studies had not been informed about an upcoming vocabulary test, nor had they been informed about the word learning aspect of the study.

Many factors have been found to have an impact on the incidental acquisition of vocabulary, such as the type of word (Kweon & Kim, 2008; Vidal, 2011), the similarity of the FL words with the NL (Vidal, 2011), and the morphological predictability of the FL words (Vidal, 2011). However, another potentially important factor is the number of exposures to the new words. Thus far, it is unclear exactly how many exposures are required before different types of word knowledge are acquired, from recognition of form to meaning recall (see Nation, 2001, for a discussion on the 12 dimensions of word knowledge). Crucially, while considerable research to date has focused on incidental learning from reading with advanced or intermediate learners of a FL (e.g., Brown, Waring, & Donkaewbua, 2008; Horst, 2005; Hulstijn, Hollander, & Greidanus, 1996; Kweon & Kim, 2008; Pellicer-Sánchez & Schmitt, 2010; Pigada & Schmitt, 2006; Rott, 1999; Vidal, 2011; Waring & Takaki, 2003), little is known about the number of exposures necessary in other contexts such as multimodal situations (a situation where the FL is provided through a combination of written and/or auditory input as well as pictorial information) or when the learners are complete beginners in a FL. In a recent study, it was found that, within 8 exposures to new words in a multimodal incidental learning situation, participants who had no prior knowledge of the language acquired FL vocabulary (Bisson, van Heuven, Conklin, & Tunney, 2013). However, can vocabulary acquisition occur from even fewer exposures to the FL words? Furthermore, if incidental acquisition of vocabulary is found to occur with fewer exposures, can we expect an increase in vocabulary acquisition commensurate with an increase in the number of exposures?

One of the first to study the effects of repeated exposure on learning and memory was Ebbinghaus (1885/1964). In a famous series of experiments, he memorized series of nonsense syllables until he could recite them. Exposure to the series of syllables during the learning phase varied between 8 and 64, and learning was achieved through reading and reciting. After the learning phase, Ebbinghaus measured the savings in relearning (a decrease in the time taken to relearn a series of syllables compared to the time taken to learn it originally) as a function of the length of the series, the retention interval, and, most relevant for this research, as a function of the number of initial exposures. Ebbinghaus found that increased exposures during the learning phase led to a decrease in the time necessary to relearn the series of syllables the next day, which is essentially a repeated exposure effect. Ebbinghaus's methods and ideas are still relevant today, and many recent studies in the field of incidental vocabulary acquisition have investigated the effects of repeated exposures on the learning and retention of vocabulary. The next section reviews some of these studies.

## Incidental Vocabulary Learning Studies

### Unimodal Input: Written Information

In the field of FL vocabulary acquisition, many studies have investigated the incidental acquisition of vocabulary through reading (e.g., Brown et al., 2008; Horst, 2005; Hulstijn et al., 1996; Kweon & Kim, 2008; Pellicer-Sánchez & Schmitt, 2010; Pigada & Schmitt, 2006; Rott, 1999; Vidal, 2011; Waring & Takaki, 2003). These studies have found that vocabulary acquisition through reading requires many exposures to the novel words in a meaningful context in order for knowledge to be acquired. For example, Pellicer-Sánchez and Schmitt (2010) asked participants to read a 150-page novel containing 34 unknown target words (the target words were in an African dialect among an English text) split into five frequency bands according to their number of occurrences in the text. Some of the target words appeared only once (10 words) while some appeared 2–4 times (10 words), 5–8 times (9 words), 10–17 times (2 words), and 28 or more times (3 words). The vocabulary gains were measured using spelling recognition, word class knowledge, meaning recognition, and meaning recall tests. Across all tests of word knowledge and all number of occurrences, the authors concluded that knowledge gains were found on about nine of the new words. Furthermore, an overall frequency of exposure effect was found, such that learners had significantly more knowledge across all the tests on the words that occurred 10 or more times in the novel compared to words that occurred 8 or fewer times. Unfortunately, because the study involved authentic text, the number of words in each frequency band could not be controlled, therefore only 5 words occurred 10 or more times, while 29 words occurred 8 or fewer times. In addition, a control group who only completed the vocabulary tests would have been useful to confirm that the vocabulary gains were due to the reading of the novel.

Rather than using a novel, Vidal (2011) asked native Spanish speakers to read a series of three university lectures in English and measured vocabulary gains using an adaptation of the Vocabulary Knowledge Scale (Paribakht & Wesche, 1997; Wesche & Paribakht, 1996). The scale included form recognition, meaning recall, NL translation, and using the word in a sentence. Vidal compared the participants’ scores prior to and following the reading of the lectures. Overall, knowledge gains were found on approximately 19 words out of 36,^1^ and the number of exposures (from one to six) was found to be a significant predictor of vocabulary gains with 47% of the variance explained by this predictor. Unfortunately, the number of words for each frequency of exposure was uneven with, for example, only 3 words occurring four times in the text compared to 10 words occurring five times. However, similar to Pellicer-Sánchez and Schmitt (2010), this study used authentic material as FL input and therefore it was not possible to control for this aspect of the material.

In contrast, in order to have more control over the number of exposures to the FL words, some studies either created their own reading material or altered existing authentic material specifically to investigate the incidental acquisition of vocabulary through reading (e.g., Hulstijn et al., 1996; Rott, 1999). For example, Rott (1999) wrote a set of short paragraphs each including novel words that participants read once per week. Using a between-subject design, participants were exposed to six target words either two, four, or six times before completing a productive vocabulary task (“supply a definition”) and a receptive vocabulary task (“select a definition”) to assess their acquisition of word knowledge. Results showed an effect of exposure frequency overall, with participants who were exposed to novel words six times consistently outperforming participants who had been exposed to the words two or four times. Furthermore, scores on items from two exposures were significantly higher than scores on control items that participants had not been exposed to. Interestingly, in contrast to the other two studies, which used advanced FL learners, participants in the Rott study were intermediate FL learners.

Further work on incidental word learning through reading has been conducted with native speakers acquiring novel pseudowords, which is similar to highly fluent learners of a FL acquiring novel FL words. The following studies investigated early learning effects using electroencephalography (EEG) to record event-related potentials (ERP). These studies focused on the N400 component. This ERP component is characterized by a negative fluctuation in the electrical brain activity happening around 400 milliseconds post stimulus presentation and has been associated with semantic processing (Kutas & Federmeier, 2000). A reduction in the N400 component has been used to demonstrate meaning integration in adult word learning studies, indicating a learning effect (e.g., Batterink & Neville, 2011; Borovsky, Kutas, & Elman, 2010; Dobel et al., 2009; McLaughlin, Osterhout, & Kim, 2004). For example, Batterink and Neville (2011) used the N400 to explore meaning integration across 10 exposures to words embedded in short stories. Overall, they found a larger reduction in the N400 for novel words with a consistent meaning compared to novel words for which no consistent meaning could be derived (hence controlling for an effect due to the repetition of the word form only). Interestingly, the difference between the two types of novel words emerged as early as the second encounter with the words.

In another study, participants read triplets of sentences where the meaning of a new word could be derived from the context (Mestres-Missé, Rodriguez-Fornells, & Münte, 2007). Remarkably, the results showed that following the third encounter during meaningful sentences, the N400 component for the new words was undistinguishable from that of known words. Further evidence of early learning effects has been found following a single encounter with a new word (Borovsky et al., 2010). Here it was found that the N400 amplitude was reduced following the plausible usage of a novel word, compared to an implausible usage, in a test sentence presented immediately after the exposure to the novel word in a meaningful context. Overall, early learning effects were found either during the exposure to the new words or immediately after the presentation of each word, and therefore it is unclear whether these effects are long lasting. It is important to note that, because participants in Borovsky et al. (2010) responded to a test sentence after each context sentence, they may have become aware of the purpose of the study early on. Once the word learning aspect of a study is revealed, learning is likely no longer incidental. The same can be said for the study by Batterink and Neville (2011), which tested vocabulary acquisition after each of four stories, thus giving away the purpose of the study after the first story. In addition, in Mestres-Missé et al. (2007), participants were specifically told to try and derive the meaning of the new words, which clearly differs from an incidental learning situation. Nevertheless, what emerges from these studies is that form–meaning links can occur from just a few exposures to a new orthographic word form in a meaningful context.

### Unimodal Input: Auditory Information

The incidental acquisition of vocabulary through listening has not been as extensively investigated. However, Vidal (2011) also studied the effect of repeated exposures while students listened to the three university lectures. In contrast to the group of students who read the lectures, frequency of exposure was not as strong a predictor for this group as it explained only 24% of the variance in vocabulary gains. Nonetheless, there were knowledge gains for about 12 words (see Endnote 1). It is worth noting that all the vocabulary tests in this study were conducted in the reading modality, while participants in the listening condition had only been exposed to the words through the listening modality. Because the learning and testing phase were in different modalities, this could have impacted word form recognition scores. Importantly, Brown et al. (2008) found no effect of frequency of exposure on the incidental acquisition of vocabulary through listening, even though vocabulary gains were assessed through a listening test. In their study, groups of participants listened to a FL story including 28 FL pseudoword targets. The target words in the story occurred between 2 and 20 times, and they were split into four frequency bands: 2–3 exposures, 7–9 exposures, 10–13 exposures, and 15–20 exposures, with seven words in each frequency band. Although participants were able to recognize on average eight new words, the number of exposures did not have an effect on recognition or recall test scores. It is important to note that this study did not include a control group or control items, which would have been useful to ensure that the results of the meaning recognition test can only be explained by incidental acquisition. Furthermore, each participant in this study also took part in a reading and a reading-while-listening condition (with a different text and target words), each of which was followed by a test session. Therefore, after the first test session, participants would have expected a vocabulary test.

### Bimodal Input: Auditory and Written Information

Another type of incidental learning situation used by reading researchers and language teachers is reading-while-listening to the same story (e.g., Brown et al., 2008; Horst, Cobb, & Meara, 1998; Webb, Newton, & Chang, 2013). This situation allows the learner to follow the written words as they listen to the pronunciation, which presumably helps them segment the seemingly uninterrupted flow of words into more manageable chunks. Brown et al. (2008) investigated the incidental acquisition of FL vocabulary in a reading-while-listening to stories situation and found that words with a higher number of occurrences in the stories were more likely to be remembered. There were significant differences in the amount of words correctly recalled and recognized between 2–3 occurrences, 7–9 occurrences, and 10–13 occurrences, but encountering the words 15–20 times did not improve the scores further. Another study investigated the incidental acquisition of collocations (i.e., multiple word units that cooccur more often than chance) through reading-while-listening to stories (Webb et al., 2013). The text of the stories was altered such that each participant was exposed to the 18 collocations either 1, 5, 10, or 15 times (between-subject design). Importantly, performances in the experimental groups were compared to a control group with no exposure to the collocations. The results showed incidental learning of the form of the collocations from 5 exposures at the receptive level and from 10 exposures at the productive level, while incidental learning of the meaning of the collocations occurred from 10 exposures at the productive level and from 15 exposures at the receptive level. Interestingly, in all tests, there were significantly greater knowledge gains in the group exposed to each collocation 15 times compared to all other groups.

The studies reviewed so far all took place with either intermediate or advanced FL learners or with native speakers learning pseudowords. These studies used reading, listening, and reading-while-listening as incidental learning situations, thus, learners had to derive the meaning of novel words from the context. With complete beginners in a FL, it would be difficult to study incidental acquisition in such situations, as learners do not possess enough knowledge to be able to infer the meaning of the words from the provided context. The exact lexical coverage necessary to infer meaning from context is still debated and seems to depend on the incidental learning situation. However, researchers tend to estimate that between 95% and 99% of the words in a text must be known in order for learners to do this (e.g., Nation, 2001; Schmitt, 2008, 2010). One way of facilitating meaning acquisition of new FL words might be to expose learners to FL input in combination with pictorial information. Webb and Rodgers (2009) suggested that in a situation including images, such as television programs, incidental learning may occur even at a lower lexical coverage. The next section reviews studies of incidental acquisition of FL vocabulary through exposure to both verbal (written and/or spoken modality) and pictorial information. This type of situation will be hereafter referred to as being multimodal.

### Multimodal Input: Written and/or Auditory Input With Pictorial Information

Although much more research has been conducted in the context of reading, listening, or reading-while-listening, a few recent studies have used multimodal situations to investigate incidental acquisition of FL vocabulary. In Bisson et al. (2013), participants were able to recognize the meaning of FL words in a translation recognition task following an incidental learning situation. This incidental learning effect emerged within eight exposures to auditory and written FL word forms presented along with a picture, even though participants had no prior knowledge of the FL. An incidental learning effect was found both immediately after exposure to the FL words, as well as the following day. Furthermore, following further explicit learning of the FL words one week later, an incidental learning advantage still occurred. It is likely that incidental learning occurred because participants were able to access the meaning of the words through the pictorial information. Because this field of research is relatively new, little is known about the number of exposures required before beginner learners can learn the form and meaning of novel words. In another recent study, Gullberg, Roberts, and Dimroth (2012) presented a 7-minute weather report in Chinese to participants without prior knowledge of Chinese. After watching the weather report either once or twice, participants completed an auditory word recognition test asking them to indicate whether Chinese words had occurred in the weather report. The number of occurrences was found to be a significant predictor of recognition scores, with words occurring more frequently in the report (8 or 16 times) recognized with significantly more accuracy than words occurring infrequently (2 or 4 times). Unfortunately, it is not clear whether the accuracy scores for each number of occurrences were significantly above chance. Furthermore, no control items were included in the study. Therefore it is difficult to determine whether the higher accuracy scores for the more frequent items were due to the number of occurrences of the items or whether the items themselves were easier to recognize.

Overall, the results of previous studies suggest that there is incremental repetition of exposure effects in the incidental acquisition of vocabulary. However, this seems to be modulated by the type or combinations of inputs in the learning situation (written and/or spoken and/or pictorial). Furthermore, the proficiency of the learners seems to play a role in incidental learning. Importantly, how many exposures are required for incidental learning to occur with beginner learners in a multimodal situation remains an open question.

Another important issue to consider when investigating incidental learning is the choice of method used to assess the vocabulary gains. In the FL incidental learning literature, most studies have used traditional recognition and recall tests. However, such tests might not tap into the very earliest stages of word learning. Very early learning effects have been detected using EEG, albeit with native speakers encountering pseudowords. In Bisson et al. (2013), a variation of the savings paradigm (see Ebbinghaus, 1885/1964, for the original savings paradigm) was used to detect traces of word knowledge that had not necessarily reached the threshold for explicit recognition. Participants were exposed to novel words in an incidental learning phase before being asked to learn words explicitly. As half of the words in the explicit learning phase had been presented in the incidental learning phase and half of the words were completely new, it was possible to determine whether simple exposure (incidental learning) impacted later overt vocabulary learning. Crucially, participants performed reliably better in the explicit learning phase for FL words they had been exposed to during the incidental learning phase compared to new FL words.

As highlighted in the discussion above, in some of the previous research there have been methodological concerns that make it problematic to draw strong conclusions about incidental vocabulary acquisition. For example, many prior studies have not made use of a control group and/or control items in order to conclude that their learning and repetition effects are due to their experimental manipulation (e.g., Pellicer-Sánchez & Schmitt, 2010; Brown et al., 2008). In some studies, learning is measured by comparing pre- and posttests (e.g., Rott, 1999; Vidal, 2011), and this can be problematic as it increases participants’ awareness of the word-learning aspect of the experiment and draws their attention to specific words. Some studies have used different numbers of words in their frequency bands (e.g., between 2 and 10 words per frequency band in Pellicer-Sánchez and Schmitt [2010] and between 3 and 10 words for each number of exposures in Vidal [2011]) while others used only a few items overall (e.g., six target words in Rott [1999]).

Most importantly, there is a lack of research on the incidental acquisition of vocabulary both in multimodal situations and with complete beginners in a FL. The purpose of the current study is to investigate the impact of repeated exposures on vocabulary acquisition with beginners, using a methodology sensitive to early vocabulary gains. Participants were exposed to the FL words in a multimodal incidental learning phase. Subsequently in an explicit learning phase, they were asked to learn FL words. Unbeknownst to participants, half of the words had been presented during the incidental learning phase, and half of the words were completely new. The number of exposures to the FL words in the incidental learning phase varied among two, four, six, and eight exposures, with 10 words for each number of exposures and two different sets of items. One set was used in the incidental learning phase (counterbalanced across participants) and the other set was used as a control comparison in the explicit learning phase. It was predicted that participants would perform better on the words they had been exposed to during the incidental learning phase compared to the new words. Furthermore, it was expected that performance in the explicit learning phase would increase with the number of exposures in the incidental learning phase.

## Method

### Participants

Seventy-eight participants took part in this experiment (mean age 20.7 years old, 60 females) and received payment or course credit for their participation.^2^ Participants were all students or staff at the University of Nottingham, and they all completed a self-reporting language background questionnaire to ensure that they were native English speakers and that they had no prior knowledge of the FL (Welsh) used in the study. We asked participants about their knowledge of other languages (e.g., subjective proficiency scores, age of first contact, years of experience) and specifically asked them whether they had prior experience with Welsh. Following this, 10 participants were excluded from the analyses for the following reasons: 3 participants were bilingual, 1 participant was not a native speaker of English, 4 participants reported having prior knowledge of Welsh (although very minimal), 1 participant reported living close to Wales and visiting the region frequently, and 1 participant did not answer the question related to prior knowledge of Welsh and was therefore excluded as a precaution. Finally, one further participant was excluded because of a technical problem during phase 2 of the experiment.

### Design and Stimuli

We used a repeated-measures design for this experiment with type of word (old and new) and number of exposures during the incidental learning phase (two, four, six, and eight exposures) as within-subject factors. The stimuli used for this experiment included the auditory and written word form of 80 Welsh words (taken from Bisson et al., 2013), as well as line drawings corresponding to the meaning of the words (Snodgrass & Vanderwart, 1980). All the words were concrete nouns and none of the words were Welsh–English cognates (see Appendix S1 in the Supporting Information online for the full list of words). The words were split into two sets, with one set of words used in phase 1 of the experiment (counterbalanced across participants) and both sets of words used in phase 2 of the experiment. The words presented during phase 1, the incidental learning phase, were considered old words and the words seen for the first time during phase 2, the explicit learning phase, were considered new words. Within each set of 40 words, the items were split into four subsets such that words were presented either twice, four times, six times, or eight times during the incidental learning phase (10 words for each number of exposures per set of word). In order to control for the fact that some words might be easier to learn than others, the words in each set were ranked according to the average percentage of accuracy score achieved across all participants in the control group of Bisson et al. (2013). This was used to match word difficulty across the number of exposures in subsets in the current study. In other words, although the words in each set were pseudorandomly assigned to each subset, we used the items’ ranking from Bisson et al. (2013) to ensure that each subset of items was not overall easier or harder to learn than the others, thereby controlling for word difficulty.

### Procedure

**Figure 1:**
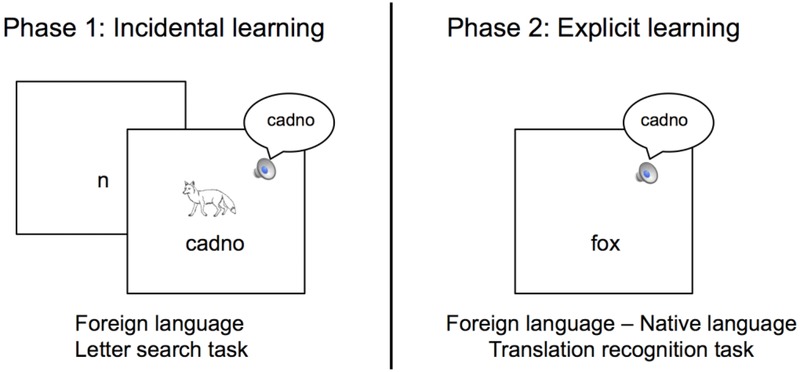
Schematic representation of phase 1 and phase 2 of the experiment.

The procedure for phases 1 and 2 of the experiment was very similar to Bisson et al. (2013). In order to provide an incidental learning situation, phase 1 of the experiment (see Figure1) consisted of a letter-search task. In this task participants were first briefly presented with a letter (500 milliseconds) prior to the appearance of a written word. Their task was to indicate with a button-press whether the letter they saw was present in the written word that would appear on the screen. For half of the trials, the letter appeared in the word (“Yes” responses) and for half of the words it did not (“No” responses). Although irrelevant for the letter-search task, participants heard the auditory form of the Welsh words and saw a line drawing depicting the meaning of the words simultaneously with the presentation of the written word forms. The presentation of the multimodal information provided an incidental learning situation in which participants could associate the meaning of the pictures with the Welsh words. Participants heard the auditory word form only once, however, the picture and the written word form remained on the screen until they made a response. Participants were not told that the FL was Welsh and they were not asked to learn the words; they were simply instructed to complete the letter-search task. Participants completed 8 practice trials with feedback prior to the main letter-search task where they completed 200 trials (stimuli were presented either twice, four times, six times, or eight times).

In phase 2 of the experiment (immediately following the completion of phase 1), participants completed a translation recognition task, in which they were explicitly asked to learn the meaning of Welsh words (see Figure1). Both sets of words (old and new) were used in phase 2, however, participants were not told that they had previously been exposed to half of the words. Participants were presented with the auditory word form of Welsh words while concurrently viewing a possible English translation. Their task was to indicate with a button-press whether the English word was the correct translation for the auditory Welsh word. Participants received feedback after each answer (“correct” or “incorrect”) and they were told to use this feedback to learn the correct translations. At the end of each block, participants were shown their percentage accuracy for the block prior to continuing to the next block. Participants were informed that their target was to reach 80% accuracy in a block of trials and that the experiment would continue for a maximum of three blocks or until they achieved 80% accuracy in one block, whichever happened first. Each auditory Welsh word was presented once with its correct English translation and once with a foil in each block (160 trials in each block). The foils were the English translations pseudorandomly assigned to an auditory Welsh word and were different for each block of trials.

## Results

### Incidental Learning: Phase 1

Accuracy was high in the letter-search task (*M* = 95%, *SE* = 0.8%) indicating that participants were attending to the written stimuli (average response time = 895 milliseconds, *SE* = 36 milliseconds). However, 1 participant only achieved 58% accuracy and was removed from further analyses.

### Explicit Learning: Phase 2

For the explicit learning phase, we report the results both by participants and by items for blocks 1 and 2. The results of block 3 were not analyzed as 14 participants reached the accuracy criterion in block 2 and therefore did not proceed to block 3. For each participant, we calculated the percentage of correct answers in the translation recognition task for the old and new words according to the number of exposures during the incidental learning phase (see Table1). These were submitted to repeated-measures analyses of variance (ANOVAs) with word type (old vs. new) and number of exposures during the incidental learning phase (two, four, six, and eight) as within-subject factors for each block separately. Results revealed a main effect of word type for both block 1, *F_1_*(1, 65) = 41.35, *p* <.001, ηp^2^ =.39, *F_2_*(1, 76) = 51.43, *p* <.001, ηp^2^ =.40, and block 2, *F_1_*(1, 65) = 23.09, *p* <.001, ηp^2^ =.26, *F_2_*(1, 76) = 28.94, *p* <.001, ηp^2^ =.28, indicating that participants performed better on old words (the words they were exposed to during the incidental learning phase) compared to new words (words occurring for the first time during block 1 of the explicit learning phase). Furthermore, for block 1, simple effects analyses revealed that accuracy scores were significantly higher for the old words for each number of exposures, *F_1_*(1, 65) = 9.05, *p* <.01, ηp^2^ =.12, *F_2_*(1, 76) = 8.10, *p* <.01, ηp^2^ =.09, *F_1_*(1, 65) = 15.97, *p* <.001, ηp^2^ =.20, *F_2_*(1, 76) = 12.28, *p* <.01, ηp^2^ =.15, *F_1_*(1, 65) = 10.99, *p* <.01, ηp^2^ =.15, *F_2_*(1, 76) = 9.62, *p* <.01, ηp^2^ =.12, *F_1_*(1, 65) = 27.81, *p* <.001, ηp^2^ =.30, *F_2_*(1, 76) = 23.92, *p* <.001, ηp^2^ =.30, for two to eight exposures during the incidental learning phase, respectively. Furthermore, the advantage gained from exposure to the words in the incidental learning phase was still significant in block 2 following further explicit learning for the words with two exposures in the incidental learning phase, *F_1_*(1, 65) = 5.13, *p* <.05, ηp^2^ =.07, *F_2_*(1, 76) = 7.42, *p* <.01, ηp^2^ =.10, six exposures, *F_1_*(1, 65) = 9.70, *p* <.01, ηp^2^ =.13, *F_2_*(1, 76) = 8.18, *p* <.01, ηp^2^ =.10, and eight exposures, *F_1_*(1, 65) = 12.83, *p* <.01, ηp^2^ =.17, *F_2_*(1, 76) = 13.52, *p* <.001, ηp^2^ =.18. For the words with four exposures in the incidental learning phase, it was no longer the case, *F_1_*(1, 65) = 2.13, *p* =.15, ηp^2^ =.03, *F_2_*(1, 76) = 2.24, *p* =.14, ηp^2^ =.05.

**Table 1:** Mean (SE) percentage of correct answers in blocks 1 and 2 of the translation recognition task (phase 2) for both old and new words according to the number of exposures during the incidental learning phase (phase 1)

	Block 1		Block 2	
	% correct		% correct	
Number of exposures	Old words	New words (control)	Old words	New words (control)
2	61.2 (1.7)	55.3 (1.5)	76.9 (1.7)	72.3 (1.7)
4	64.9 (1.6)	57.7 (1.3)	74.1 (1.7)	71.6 (1.6)
6	66.4 (1.6)	59.9 (1.5)	77.0 (1.4)	72.2 (1.4)
8	68.9 (1.3)	58.8 (1.4)	75.9 (1.5)	69.8 (1.2)

Note: None of the new words have been presented during the incidental learning phase. However, they were split into number of exposures subsets in order to provide a control comparison for the purpose of the analysis only.

**Figure 2:**
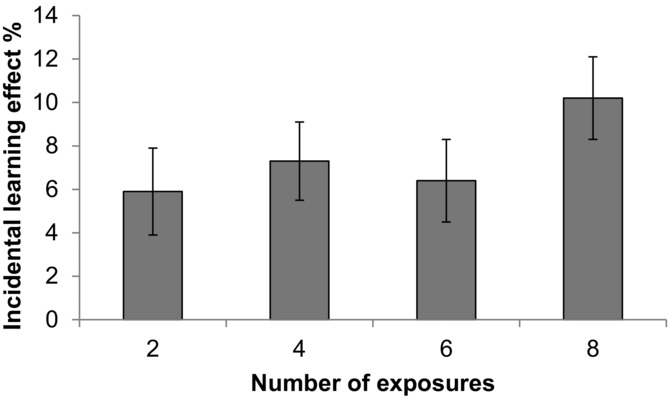
Incidental learning effect (difference score between old and new words) in block 1 of the explicit learning phase (phase 2) according to the number of exposures during the incidental learning phase (phase 1). The error bars show the standard error of the means.

In order to investigate the effect of repeated exposure, we calculated a difference score between old and new words, which we refer to hereafter as the incidental learning effect (see Figure2). The effect of repeated exposure was investigated by computing linear contrasts using a repeated-measures ANOVA in the participant analysis and a one-way ANOVA in the item analysis. Results revealed no significant linear contrasts either for block 1, *F_1_*(1, 65) = 2.31, *p* =.13, ηp^2^ =.03, *F_2_*(3, 76) = 1.64, *p* =.20, ηp^2^ =.02, or block 2, *F*s < 1. To further investigate whether an increase in the number of exposures led to an increase in incidental learning effect, we compared the incidental learning effect for the minimum and maximum number of exposures, that is, two and eight, using a paired sample *t* test and an independent sample *t* test in the participant and item analyses, respectively (one-tailed). For block 1, results revealed a significant increase in the incidental learning effect between two and eight exposures in the participant analysis, *t_1_*(65) = 1.71, *p* <.05, *d* = 0.21, and a strong trend in the item analysis, *t_2_*(38) = 1.55, *p* =.06, *d* = 0.49. However, no significant differences were found in block 2, *t*s < 1.

**Figure 3:**
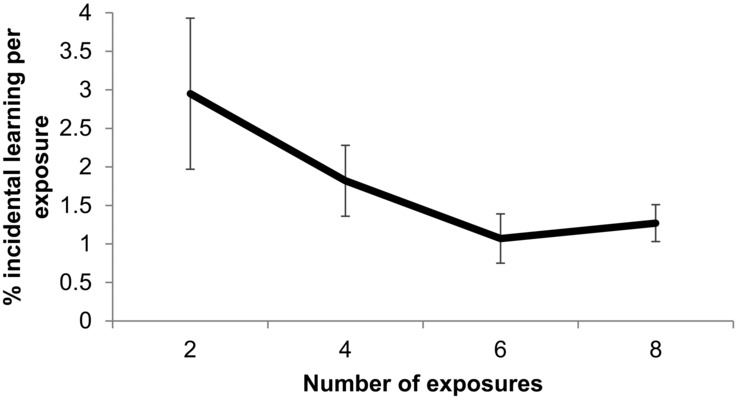
Normalized incidental learning effect for each number of exposures during the incidental learning phase (incidental learning effect divided by the number of exposures) with error bars showing the standard error of the means.

It is also possible that the knowledge gained for each exposure to the words during the incidental learning phase is not constant for each number of exposures. For example, for the words with two exposures in the incidental learning phase, accuracy scores were about 6% higher for old words compared to new words, which is equivalent to a 3% increase in accuracy per exposure to the words. In contrast, for the words with eight exposures in the incidental learning phase, the accuracy scores were approximately 10% higher for the old words, which is equivalent to a 1.25% increase per exposure. We therefore normalized the incidental learning effect by dividing the incidental learning effect by the number of exposures (see Figure3). The normalized incidental learning effects were then investigated using ANOVAs, which revealed a small but significant negative linear trend, *F_1_* (1, 65) = 3.98, *p* =.05, ηp^2^ =.06, *F_2_* (1, 76) = 4.62, *p* <.05, ηp^2^ =.05, indicating that the knowledge gained from each exposure decreased as the number of exposures increased. As this data were not normally distributed, we also computed Pages for *F_1_* and Jonckheere for *F_2_* analyses of trend tests. The results again revealed significant linear trends in both cases, *L* = 1692, *z* = 2.03, *p* <.05, *r* =.25, *J* = 994, *z* = 1.78, *p* <.05, *r* =.20.

## Discussion

The aim of the current study was to investigate the effect of repeated exposures to multimodal stimuli on the incidental acquisition of FL vocabulary. The results replicated the previous findings of Bisson et al. (2013), demonstrating an incidental learning effect on the performance in the translation recognition task with participants without prior knowledge of the FL. Furthermore, the incidental learning effect persisted following further explicit learning in block 1 into block 2. Crucially, the current results showed that even when participants were only exposed to stimuli twice in the incidental learning phase, their performance improved in the explicit learning phase. This indicates that knowledge about the form and/or meaning of the lexical items was acquired after as little as two exposures to multimodal stimuli in an incidental learning situation.

Importantly, the results suggest that more exposures in the incidental learning phase led to better performance on the translation recognition task. However, this was only the case when comparing the scores from the minimum and maximum number of repetitions (two versus eight exposures). Although we did not find an overall effect of repeated exposures, further analyses showed that this was likely due to the fact that the impact of each encounter with a word in the incidental learning phase was not constant across number of exposures. Crucially, as the number of exposures increased, additional encounters with a word had less of an impact on incidental learning.

It is important to consider that, in contrast to reading, listening and reading-while-listening situations, in which the meaning of the words must be derived from the context of a sentence, the meaning of the words in the current study could easily be ascertained from the pictorial stimuli. In addition, the pictures used were simple static line drawings, the FL words were presented as isolated words, and the words were always presented with the same line drawing. Taking all this into account, it is likely that meaning was extracted from these representations early on. This could explain why further encounters had less of an impact. It is also likely that form–meaning links are easier to establish when both form and meaning are presented simultaneously or contiguously. Thus, there may be an advantage to being exposed to FL words through a multimodal situation with pictorial information, because in this situation, word meaning can be presented clearly and at the same time as the word forms. In a more complex multimodal situation, for example, a film with subtitles, where the meaning of the words is less transparent and where the words are presented in sentences rather than isolated words, it is likely that more repetitions will be necessary for knowledge to be acquired (e.g., Bisson, van Heuven, Conklin, & Tunney, 2014). Furthermore, the words used in the current study were all concrete nouns and such words have been shown to be easier to learn than, for example, abstract nouns (e.g., de Groot & Keijzer, 2000). Therefore, although in this study we found learning effects from two exposures, this may not be enough in more complex contexts or when the meaning of the novel words is less clear. In addition, more exposure may be required with other types of words such as abstract nouns, verbs, or adjectives.

Another factor that may have influenced the unequal impact of additional exposures to novel FL in the current study is the incidental learning phase itself. Here participants had to complete a letter-search task, and this did not require them to pay attention to the FL auditory word forms or to the pictures; only the FL written word forms were relevant for the task. Thus, initially participants may have been interested in all the stimuli because of their novelty and saliency. However, after the first few exposures, they might no longer have been interested in attending to this extra information. It would therefore be important in future research to use eye tracking to investigate the allocation of attention to the pictorial and written stimuli and, more specifically, to explore the pattern of fixations across repetitions of the stimuli to determine how this impacts acquisition. Furthermore, participants responded faster on average to the words that were repeated more often in the incidental learning phase.^3^ Therefore, they spent less time on the stimuli during each exposure as the number of exposures increased, impacting the learning gains. Future experiments should attempt to disentangle the effect of repeated exposures versus the effect of the duration of exposure.

An overall effect of repeated exposure has been found in other contexts such as reading (Brown et al., 2008; Hulstjin et al., 1996; Pellicer-Sánchez & Schmitt, 2010; Rott, 1999; Vidal, 2011), listening (Vidal, 2011), and reading-while-listening (Brown et al., 2008; Horst et al., 1998; Webb et al., 2013). However, the effect is difficult to compare across studies, as varied numbers of exposures were used. A normalized incidental learning effect, as was used in the current study, might therefore be useful to compare results across studies and to ascertain whether the unequal impact of additional exposures found in the current study is generalizable to other contexts. Furthermore, not many studies reported the minimum number of exposures required to detect a significant incidental learning effect. To our knowledge, the two exceptions are Webb et al. (2013), who found significant incidental learning from five exposures compared to a control group that was not exposed to the words, and Rott (1999), who found an earlier effect, with two exposures leading to significant incidental learning compared to a control set of items without prior exposure. It would therefore be useful in the future that studies investigating the impact of the number of exposures on incidental acquisition of vocabulary also include comparisons with a control group or a set of control items.

## Limitations and Conclusion

The results of the current study indicate that the incidental acquisition of vocabulary can happen extremely fast even with complete beginners in a FL. As little as two exposures to new words in a multimodal incidental learning situation was enough for knowledge about the new words to be acquired. In addition, we found that the impact of exposure was not constant across number of exposures, but rather decreased following the initial encounters. Overall, our findings suggest that very few exposures to new words are required before learning starts to occur. This finding is very encouraging for language teachers, as an incidental learning activity could easily be included at the beginning of a lesson to introduce new (concrete, depictable) vocabulary. The results of the current study showed that very few exposures are required to impact subsequent explicit learning. Whereas the current study used a letter-search task as an incidental learning activity, any activity that encourages exposure to FL word forms and meanings could be used by language teachers. For example, teachers could use a word game where the focus of the activity is on the word forms or grammatical features of the words. Providing a picture with each word (even though it is not necessary for the task) would promote the incidental learning of word meaning. Importantly, this type of activity is not limited to the language classroom and could be carried out as an extracurricular activity.

The variation on the savings paradigm used in the current study was sensitive enough to detect vocabulary acquisition from two exposures to FL words. However, a limitation of this paradigm is that it is not possible to determine whether the knowledge acquired during the incidental learning phase was purely below the recognition threshold or at the level of recognition. Furthermore, because participants received feedback during the explicit learning phase and used this to continue acquiring knowledge about the words, we cannot draw concrete conclusions about the type of knowledge that was acquired specifically in the incidental learning phase. However, what is important is that some knowledge was acquired during the incidental learning phase that improved the subsequent explicit learning. Further, as discussed in Bisson et al. (2013), it is unclear whether this initial word knowledge was linked directly to semantic representations (accessed through the pictorial information) or whether participants activated NL word representations when they processed the pictorial information, which they in turn linked to the FL word forms. Another important consideration is that our translation recognition measure did not allow us to detect knowledge past the recognition of the initial form–meaning link. It would therefore be useful to assess further learning in the future using, for example, a recall task. Furthermore, it will be important to investigate the impact of different numbers of exposures following a time delay, as in the current study, both incidental and explicit learning phases occurred within the same session. Although an incidental learning effect was found in Bisson et al. (2013) following both a day and a week delay, the study did not investigate the impact of repeated exposures. Finally, our fast learning effect is consistent with studies showing that two exposures to novel words while reading led to a learning effect (Rott, 1999), as well as to previous EEG studies which tap into early stages of word learning (see Batterink & Neville, 2011; Borovsky et al., 2010; Mestres-Missé et al., 2007).

Taken together, our results suggest that studies investigating incidental vocabulary acquisition would benefit from using much more sensitive measuring instruments, such as the variation of the saving paradigm used in the current study. Furthermore, in order to better evaluate the impact of the number of exposures, researchers should take into account that each encounter with new words does not necessarily lead to the same amount of learning.

## Notes

1 This number was estimated using Figure2 of Vidal (2011).

2 Three participants did not provide age information and 2 participants did not indicate their gender.

3 We calculated the average response times for participants during the incidental learning phase for the FL words presented for each number of exposures. Participants took an average of 883 milliseconds (*SE* = 24 milliseconds) to respond to the stimuli presented twice, 847 milliseconds (*SE* = 23 milliseconds) to respond to the stimuli presented four times, 860 millisecond (*SE* = 22 milliseconds) to respond when stimuli were repeated six times and 827 milliseconds (*SE* = 22 milliseconds) to respond when stimuli were repeated eight times. Participants responded increasingly faster to stimuli that were presented more often, as revealed by a significant linear contrast in a repeated-measures ANOVA, *F_1_*(1, 65) = 44.54, *p* <.001, ηp^2^ =.41, *F_2_*(1, 76) = 5.46, *p* <.05, ηp^2^ =.07.

## Supporting Information

Disclaimer: Supplementary materials have been peer-reviewed but not copyedited.

Appendix S1: List of Experimental Words.

## References

[b1] Batterink L, Neville H (2011). Implicit and explicit mechanisms of word learning in a narrative context: An event-related potential study. Journal of Cognitive Neuroscience.

[b2] Bisson M-J, van Heuven WJB, Conklin K, Tunney RJ (2013). Incidental acquisition of foreign language vocabulary through brief multi-modal exposure. PLoS ONE.

[b3] Bisson M-J, van Heuven WJB, Conklin K, Tunney RJ (2014). Processing of native and foreign language subtitles in films: An eye tracking study. Applied Psycholinguistics.

[b4] Borovsky A, Kutas M, Elman J (2010). Learning to use words: Event-related potentials index single-shot contextual word learning. Cognition.

[b5] Brown R, Waring R, Donkaewbua S (2008). Incidental vocabulary acquisition from reading, reading-while-listening, and listening to stories. Reading in a Foreign Language.

[b6] de Groot AMB, Keijzer R (2000). What is hard to learn is easy to forget: The roles of word concreteness, cognate status, and word frequency in foreign-language vocabulary learning and forgetting. Language Learning.

[b7] de Groot AMB, de Groot AMB, van Hell JG, Kroll JF (2005). The learning of foreign language vocabulary. Handbook of bilingualism: Psycholinguistic approaches.

[b8] Dobel C, Junghöfer M, Breitenstein C, Klauke B, Knecht S, Pantev C (2009). New names for known things: On the association of novel word forms with existing semantic information. Journal of Cognitive Neuroscience.

[b9] Ebbinghaus H, Bussenius CE, Ruger HA (1964). Memory: A contribution to experimental psychology.

[b10] Gass S (1999). Incidental vocabulary learning. Studies in Second Language Acquisition.

[b11] Gullberg M, Roberts L, Dimroth C (2012). What word-level knowledge can adult learners acquire after minimal exposure to a new language. The International Review of Applied Linguistics in Language Teaching.

[b12] Horst M (2005). Learning L2 vocabulary through extensive reading: A measurement study. Canadian Modern Language Review.

[b13] Horst M, Cobb T, Meara P (1998). Beyond a clockwork orange: Acquiring second language vocabulary through reading. Reading in a Foreign Language.

[b14] Hulstijn JH, Robinson P (2001). Intentional and incidental second language vocabulary learning: A reappraisal of elaboration, rehearsal and automaticity. Cognition and second language instruction.

[b15] Hulstijn J, Hollander M, Greidanus T (1996). Incidental vocabulary learning by advanced foreign language students: The influence of marginal glosses, dictionary use and reoccurrence of unknown words. Modern Language Journal.

[b16] Kutas M, Federmeier K (2000). Electrophysiology reveals semantic memory use in language comprehension. Trends in Cognitive Sciences.

[b17] Kweon S, Kim H (2008). Beyond raw frequency: Incidental vocabulary acquisition in extensive reading. Reading in a Foreign Language.

[b18] Laufer B (2001). Incidental vocabulary acquisition in a second language: The construct of task-induced involvement. Applied Linguistics.

[b19] McLaughlin J, Osterhout L, Kim A (2004). Neural correlates of second-language word learning: Minimal instruction produces rapid change. Nature Neuroscience.

[b20] Mestres-Missé A, Rodriguez-Fornells A, Münte TF (2007). Watching the brain during meaning acquisition. Cerebral Cortex.

[b21] Nation ISP (2001). Learning vocabulary in another language.

[b22] Paribakht ST, Huckin TN, Wesche M, Coady J (1997). Vocabulary enhancement activities and reading for meaning in second language vocabulary acquisition. Second language vocabulary acquisition: A rationale for pedagogy.

[b23] Pellicer-Sánchez A, Schmitt N (2010). Incidental vocabulary acquisition from an authentic novel: Do things fall apart. Reading in a Foreign Language.

[b24] Perruchet P, Pacton S (2006). Implicit learning and statistical learning: One phenomenon, two approaches. Trends in Cognitive Sciences.

[b25] Pigada M, Schmitt N (2006). Vocabulary acquisition from extensive reading: A case study. Reading in a Foreign Language.

[b26] Reber AS (1989). Implicit learning and tacit knowledge. Journal of Experimental Psychology: General.

[b27] Rebuschat P, Williams JN (2011). Implicit and explicit knowledge in second language acquisition. Applied Psycholinguistics.

[b28] Rott S (1999). The effect of exposure frequency on intermediate language learners’ incidental vocabulary acquisition and retention through reading. Studies in Second Language Acquisition.

[b29] Schmitt N (2008). Review article: Instructed second language vocabulary learning. Language Teaching Research.

[b30] Schmitt N (2010). Researching vocabulary: A vocabulary research manual.

[b31] Snodgrass JG, Vanderwart M (1980). A standardized set of 260 pictures: Norms for name agreement, image agreement, familiarity, and visual complexity. Journal of Experimental Psychology. Human Learning and Memory.

[b32] Vidal K (2011). A comparison of the effects of reading and listening on incidental vocabulary acquisition. Language Learning.

[b33] Waring R, Takaki M (2003). At what rate do learners learn and retain new vocabulary from reading a graded reader. Reading in a Foreign Language.

[b34] Webb S, Newton J, Chang A (2013). Incidental learning of collocation. Language Learning.

[b35] Webb S, Rodgers MPH (2009). Vocabulary demands of television programs. Language Learning.

[b36] Wesche M, Paribakht S (1996). Assessing second language vocabulary knowledge: Depth versus breath. Canadian Modern Language Review.

[b37] Williams JN (2005). Learning without awareness. Studies in Second Language Acquisition.

[b38] Williams JN, Ritchie WC, Bhatia TK (2009). Implicit learning in second language acquisition. The new handbook of second language acquisition.

[b39] Williams JN (2010). Initial incidental acquisition of word order regularities: Is it just sequence learning. Language Learning.

