# Supplementary material for: The Role of Repeated Exposure to Multimodal Input in Incidental Acquisition of Foreign Language Vocabulary
Source: Lang Learn. 2014 Oct 15;64(4):855–77. doi: 10.1111/lang.12085 (PMC4277705; doi:10.1111/lang.12085)
Supplement: Supplementary file 1 — Appendix S1: List of Experimental Words. [file lang0064-0855-sd1.docx]

**Appendix S1: List of Experimental Words**

List of English and Welsh words used with set number, number of exposures during the incidental learning phase and picture number (see Snodgrass, J. G., & Vanderwart, M. [1980]. A standardized set of 260 pictures: Norms for name agreement, image agreement, familiarity, and visual complexity. *Journal of Experimental Psychology. Human Learning and Memory*, *6*, 174–215).

| English | Welsh | Set 1 | Set 2 | Number of exposures | Picture number |
| --- | --- | --- | --- | --- | --- |
| ant | morgrug | new | old | 2 | 5 |
| barn | wen | new | old | 2 | 17 |
| bed | gwely | new | old | 2 | 22 |
| bird | adar | new | old | 2 | 28 |
| broom | ysgub | new | old | 2 | 37 |
| glove | faneg | new | old | 2 | 106 |
| leaf | deilen | new | old | 2 | 133 |
| moon | lleuad | new | old | 2 | 146 |
| nose | trwyn | new | old | 2 | 155 |
| rooster | ceiliog | new | old | 2 | 191 |
| ear | clust | new | old | 4 | 83 |
| envelope | amlen | new | old | 4 | 85 |
| foot | troed | new | old | 4 | 94 |
| fox | cadno | new | old | 4 | 98 |
| grapes | grawnwin | new | old | 4 | 109 |
| hand | llaw | new | old | 4 | 115 |
| house | ty | new | old | 4 | 122 |
| sandwich | brechdan | new | old | 4 | 195 |
| snake | neidr | new | old | 4 | 209 |
| vase | cawg | new | old | 4 | 246 |
| bell | gloch | new | old | 6 | 25 |
| box | blwch | new | old | 6 | 35 |
| carrot | moron | new | old | 6 | 48 |
| eye | llygad | new | old | 6 | 86 |
| hammer | morthwyl | new | old | 6 | 114 |
| harp | telyn | new | old | 6 | 117 |
| knife | cyllell | new | old | 6 | 130 |
| nail | hoelen | new | old | 6 | 151 |
| refrigerator | oergell | new | old | 6 | 185 |
| shirt | crys | new | old | 6 | 203 |
| beetle | chwilen | new | old | 8 | 24 |
| bow | cwlwm | new | old | 8 | 33 |
| deer | ceirw | new | old | 8 | 71 |
| dog | ci | new | old | 8 | 73 |
| flower | blodyn | new | old | 8 | 91 |
| rabbit | cwningen | new | old | 8 | 182 |
| spider | copyn | new | old | 8 | 212 |
| spoon | llwy | new | old | 8 | 215 |
| sun | haul | new | old | 8 | 222 |
| turtle | crwban | new | old | 8 | 244 |
| arm | braich | old | new | 2 | 7 |
| bee | gwenyn | old | new | 2 | 23 |
| caterpillar | lindysyn | old | new | 2 | 50 |
| duck | hwyaden | old | new | 2 | 81 |
| frog | broga | old | new | 2 | 100 |
| glass | gwydr | old | new | 2 | 104 |
| lock | clo | old | new | 2 | 143 |
| seal | selnod | old | new | 2 | 201 |
| sheep | dafad | old | new | 2 | 202 |
| watch | oriawr | old | new | 2 | 250 |
| airplane | awyren | old | new | 4 | 2 |
| axe | bwyell | old | new | 4 | 12 |
| boot | cist | old | new | 4 | 31 |
| bread | bara | old | new | 4 | 36 |
| fish | pysgodyn | old | new | 4 | 89 |
| hair | gwallt | old | new | 4 | 113 |
| heart | calon | old | new | 4 | 119 |
| stove | popty | old | new | 4 | 219 |
| table | bwrdd | old | new | 4 | 226 |
| tree | coeden | old | new | 4 | 241 |
| arrow | saeth | old | new | 6 | 8 |
| horse | ceffyl | old | new | 6 | 121 |
| leg | coes | old | new | 6 | 134 |
| mountain | mynydd | old | new | 6 | 148 |
| mouse | llygoden | old | new | 6 | 149 |
| mushroom | madarchen | old | new | 6 | 150 |
| necklace | cadwyn | old | new | 6 | 153 |
| shoe | esgid | old | new | 6 | 204 |
| thumb | bawd | old | new | 6 | 231 |
| wheel | olwyn | old | new | 6 | 254 |
| barrel | gasgen | old | new | 8 | 18 |
| chair | cadair | old | new | 8 | 53 |
| comb | crib | old | new | 8 | 65 |
| finger | bys | old | new | 8 | 88 |
| fly | pryf | old | new | 8 | 93 |
| gun | dryll | old | new | 8 | 112 |
| hanger | cambren | old | new | 8 | 116 |
| kettle | tegell | old | new | 8 | 127 |
| pig | mochyn | old | new | 8 | 172 |
| snail | malwen | old | new | 8 | 208 |
